# Supplementary material for: Complexome profiling of the Chlamydomonas psb28 mutant reveals TEF5 as an early PSII assembly factor
Source: Plant Cell. 2025 Mar 17;37(6):koaf055. doi: 10.1093/plcell/koaf055 (PMC12164590; doi:10.1093/plcell/koaf055)
Supplement: koaf055_Supplementary_Data [file koaf055_supplementary_data.zip › psb28_interactive.zip!/plots/intensity/qualityPlots/peptides/CGL59_Cre06.g280650.t1.2.html]

### Cre06.g280650.t1.2

Trivial name: CGL59  
SeqCoverage [median]: 52.60  
Mass: 29.37 kDa  
  
 

| Sequence | MissedCleavage | Mass [Da] | wtIntensity | muIntensity | Score |
| --- | --- | --- | --- | --- | --- |
| AQDIEDER | 0 | 974.43 | 902.10 | 598.28 | 111.17 |
| AQDIEDERGVSR | 1 | 1373.65 | 723.39 | 0.00 | 64.64 |
| AQDIEDERGVSRDEMAQALIEVGEGR | 2 | 2872.36 | 1732.75 | 3020.29 | 59.43 |
| ATSEEVDLEVQVEQFMKR | 1 | 2137.04 | 0.00 | 11220.25 | 89.98 |
| DEMAQALIEVGEGR | 0 | 1516.72 | 2480.69 | 3446.39 | 117.20 |
| DEMAQALIEVGEGRVPNDR | 1 | 2098.01 | 0.00 | 1701.83 | 40.86 |
| DLATIIGGDVVSEEMAQR | 0 | 1902.94 | 5937.37 | 0.00 | 162.59 |
| DMSFNEVK | 0 | 968.43 | 8878.93 | 19075.16 | 178.30 |
| GTVAAAPAAPAKPSASDYASLMGGDTVVKPYVMGDNLR | 2 | 3747.86 | 17506.81 | 26172.49 | 70.52 |
| GVSRDEMAQALIEVGEGR | 1 | 1915.94 | 492.75 | 1660.53 | 71.70 |
| LIVSIEDPR | 0 | 1040.59 | 47122.49 | 120698.02 | 202.45 |
| LIVSIEDPRAR | 1 | 1267.72 | 0.00 | 617.80 | 41.28 |
| QAEIESGAAFVRPK | 1 | 1501.79 | 40462.17 | 55891.10 | 188.13 |
| RAQDIEDER | 1 | 1130.53 | 0.00 | 793.05 | 76.94 |
| TRDMSFNEVK | 1 | 1225.58 | 5667.35 | 10674.79 | 128.01 |
|  |  |  | 131906.8 | 255570.0 |  |
